# Regulation of DNA transposition by CpG methylation and chromatin structure in human cells

**DOI:** 10.1186/1759-8753-4-15

**Published:** 2013-05-15

**Authors:** Tobias Jursch, Csaba Miskey, Zsuzsanna Izsvák, Zoltán Ivics

**Affiliations:** 1 Max Delbrück Center for Molecular Medicine, D-13125, Berlin, Germany; 2 Division of Medical Biotechnology, Paul Ehrlich Institute, Paul Ehrlich Str. 51-59, D-63225, Langen, Germany

## Abstract

**Background:**

The activity of transposable elements can be regulated by different means. DNA CpG methylation is known to decrease or inhibit transpositional activity of diverse transposons. However, very surprisingly, it was previously shown that CpG methylation of the *Sleeping Beauty* (*SB*) transposon significantly enhanced transposition in mouse embryonic stem cells.

**Results:**

In order to investigate the unexpected response of *SB* transposition to CpG methylation, related transposons from the *Tc1*/*mariner* superfamily, that is, *Tc1*, *Himar1*, *Hsmar1*, *Frog Prince* (*FP*) and *Minos* were tested to see how transposition was affected by CpG methylation. A significant increase of >20-fold in transposition of *SB*, *FP* and *Minos* was seen, whereas *Tc1*, *Himar1* and *Hsmar1* showed no difference in transposition upon CpG-methylation. The terminal inverted repeats (TIRs) of the *SB*, *FP* and *Minos* elements share a common structure, in which each TIR contains two functionally important binding sites for the transposase (termed the IR/DR structure). The group of IR/DR elements showed increased excision after CpG methylation compared to untreated transposon donor plasmids. We found that *de novo* CpG methylation is not required for transposition. A mutated *FP* donor plasmid with depleted CpG sites in both TIRs was as efficient in transposition as the wild-type transposon, indicating that CpG sites inside the TIRs are not responsible for altered binding of factors potentially modulating transposition. By using an *in vivo* one-hybrid DNA-binding assay in cultured human cells we found that CpG methylation had no appreciable effect on the affinity of *SB* transposase to its binding sites. However, chromatin immunoprecipitation indicated that CpG-methylated transposon donor plasmids are associated with a condensed chromatin structure characterized by trimethylated histone H3K9. Finally, DNA compaction by protamine was found to enhance *SB* transposition.

**Conclusions:**

We have shown that DNA CpG methylation upregulates transposition of IR/DR elements in the *Tc1/mariner* superfamily. CpG methylation provokes the formation of a tight chromatin structure at the transposon DNA, likely aiding the formation of a catalytically active complex by facilitating synapsis of sites bound by the transposase.

## Background

Co-evolution of transposable elements (TEs) with their host species gave rise to several mechanisms that regulate the transposition reaction [1], for example, cell or tissue type [2,3], cell-cycle timing [4], transcriptional regulation [5-7], posttranscriptional regulation by small RNAs [8-10], regulation of the transposase protein [11], interactions with DNA repair [12], and target site selectivity. The activity of TEs can be regulated by chromatin at different stages of the transpositional reaction. For example, the formation of a catalytically active synaptic complex requires expression of the transposase and a DNA topology that makes the element accessible for the protein machinery required for catalysis. The eukaryotic genome is typically organized into either of two types of chromatin: euchromatin, a relatively relaxed chromatin structure, in which the DNA is packed less tightly and heterochromatin, a more inaccessible and highly condensed fraction of the genome. Heterochromatic regions carry characteristic features, which distinguish them from euchromatic DNA, such as dense cytosine-methylation (5-Me-C) of CpG sites, hypo-acetylation of lysine residues in the N-terminal tails of histones H3 and H4 and methylation of specific lysine residues such as lysine 9 in histone H3. In contrast to euchromatin, which is largely composed of unique (protein coding) sequences, the DNA sequence of heterochromatin is usually repetitive and gene poor [13]. One class of repetitive sequences found in heterochromatic regions of different genomes are TEs, and therefore it is believed that the accumulation of transposable DNA sequences in heterochromatic regions provides a safe place, where the deleterious potential of these elements can be kept on a leash [14]. Indeed, there is a strong correlation between chromatin structure and the activity of TEs. For example, recruiting transposable DNAs into heterochromatic regions may provide efficient silencing of transcription of element-encoded proteins, and thus provides genome stability. In addition to its repressive function on transcription, heterochromatin also exerts a repressive influence on recombination [15,16]; hence, the containment of repeated sequences in heterochromatic regions may prevent irregular recombination and genome instability.

CpG methylation is known to decrease or inhibit transpositional activity of diverse transposons. However, very surprisingly, Yusa *et al*. showed that CpG methylation of the *Sleeping Beauty* (*SB*) transposon and to a smaller extent the *Tc3* element of *Caenorhabditis elegans* produced elevated transpositional activity in mouse embryonic stem (ES) cells [17]. Chromatin immunoprecipitation experiments revealed that hyperactive genomic donor sites have the characteristics of a heterochromatic structure. The *SB* transposase was found to co-localize with heterochromatin protein 1 (HP1), a well-established marker for heterochromatin, suggesting that the transposase preferentially associates with heterochromatic DNA [18]. Based on these results, it was postulated that heterochromatin formation at the transposon donor site can upregulate *SB* transposition [17].

**Figure 1:**
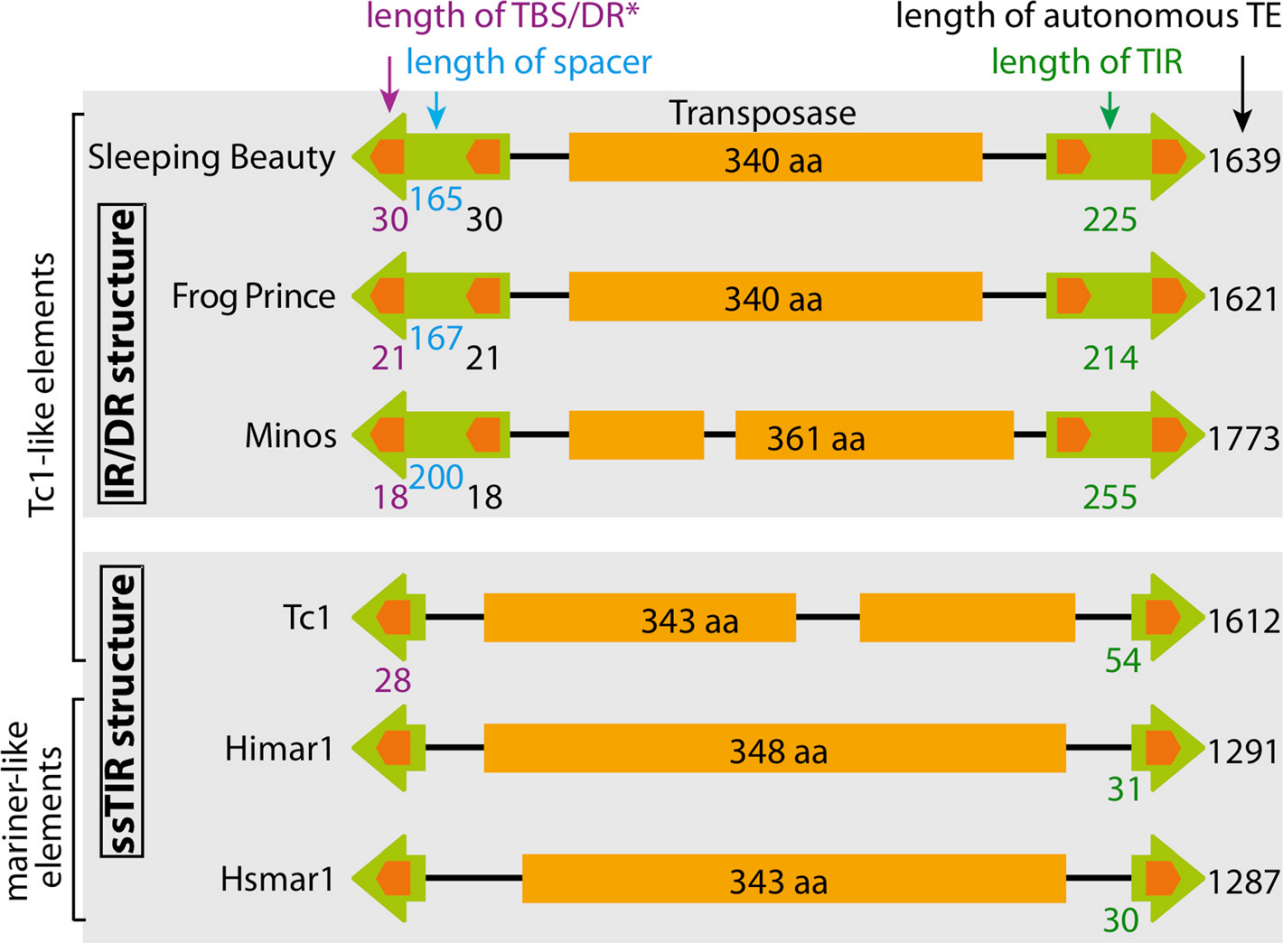
**Structure of *Tc1*/*mariner *transposons.** The transposons can be grouped by their different TIR structure. IR/DR TEs have long TIRs with two transposase binding sites (TBSs) per TIR in the same orientation (direct repeats). Simple-structured TEs have short TIRs with only one TBS. *Frog Prince* has 26 bp-long DRs, but the TBSs are only 21 bp. DR: direct repeat; ssTIR: simple-structured TIR; TBS: transposase binding site; TE: transposable element; TIR: terminal inverted repeat; IR/DR: inverted repeat/direct repeat.

We addressed the question whether transposition of other *Tc1/mariner* elements is also enhanced by CpG methylation. The elements of the *Tc1*/*mariner* superfamily can be subdivided into two groups based on the size and structure of their terminal inverted repeats (TIRs) (Figure 1) [19]. The first group is characterized by short TIRs (approximately 25 bp to 60 bp) containing a single transposase binding site (TBS) per TIR, whereas the second group has longer TIRs (>200 bp) harboring two TBSs in the same orientation, which are thus referred to as direct repeats (DRs). This arrangement is called the IR/DR structure [19]. Here we extend the original observations on *SB* transposition by examining the effects of CpG methylation on transposition of other *Tc1*/*mariner* elements. Transposition of six different transposons: *SB*, *Frog Prince* (*FP*) [20], *Minos*[21], *Tc1*[22], *Himar1*[23] and *Hsmar1*[24] was analyzed. We found that the enhancing effect of CpG methylation is not restricted to *SB*, but does not universally apply within the *Tc1*/*mariner* family either. A shared feature of *SB*, *FP* and *Minos* transposon TIRs is the IR/DR structure. We propose a model where formation of a condensed chromatin structure at transposon sequences is responsible for the observed increase in transposition.

## Results

### Transpositional activities of different *Tc1/mariner* elements upon CpG methylation

**Figure 2:**
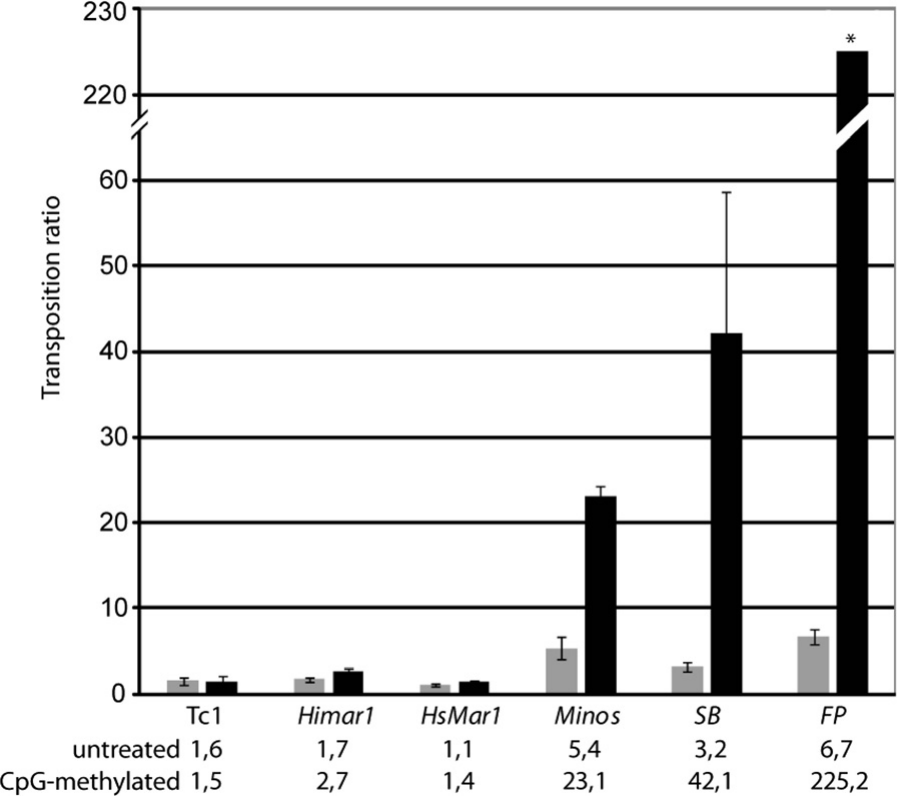
**Transposition activity of *Tc1/mariner *elements upon CpG methylation.** CpG-methylated (black bars) or untreated (grey) *Tc1*-, *Himar1*-, *Hsmar1*-, *Minos*-, *SB*-, *FP*-gene trap donor plasmids were co-transfected with/without transposase into HeLa cells and selected with G418. The transposition ratio was calculated by dividing the numbers of G418-resistant colonies obtained in the presence of transposase by the number of colonies obtained in the absence of transposase. Error bars are the standard deviation; the asterisk indicates that the error bar cannot be displayed for *FP* (±38.6). *FP*: *Frog Prince*; *SB*: *Sleeping Beauty.*

It was previously found that *SB* transposition is enhanced by CpG methylation [17]. In order to extend these observations to other *Tc1*/*mariner* transposons, the effect of CpG methylation on *Tc1* and the *Tc1*-like elements *FP* and *Minos*, and the *mariner*-like elements *Himar1* and *Hsmar1* was investigated. We generated transposon donor plasmids containing the respective TIRs flanking a neomycin-resistance gene trap cassette cloned into identical plasmid backbones. Because CpG methylation would silence a transgene promoter (thereby compromising the selection of antibiotic-resistant cell colonies upon transposition), we utilized a splice acceptor site upstream of the neomycin-resistance gene, so that expression of the selectable marker is dependent on transposition into an endogenous, expressed gene. The donor plasmids were CpG-methylated *in vitro*, and methylated or untreated donor plasmids were co-transfected with the respective transposase-expressing helper plasmids into cultured HeLa cells and selected with G418. Confirming previous findings [17], transposition of *SB* was enhanced >tenfold upon CpG methylation (Figure 2). Varying degrees of enhancement of transposition were also seen for the *FP* and *Minos* elements; the greatest increase in transposition, >30-fold, was seen for the *FP* element (Figure 2). In contrast, transposition of *Tc1*, *Himar1* and *Hsmar1* was not or only weakly affected by CpG methylation (Figure 2). These data indicate that *Tc1* family transposons of the IR/DR structure group are responsive to CpG methylation.

### CpG methylation does not affect binding of the *Sleeping Beauty* transposase to transposon inverted repeats *in vivo*

**Figure 3:**
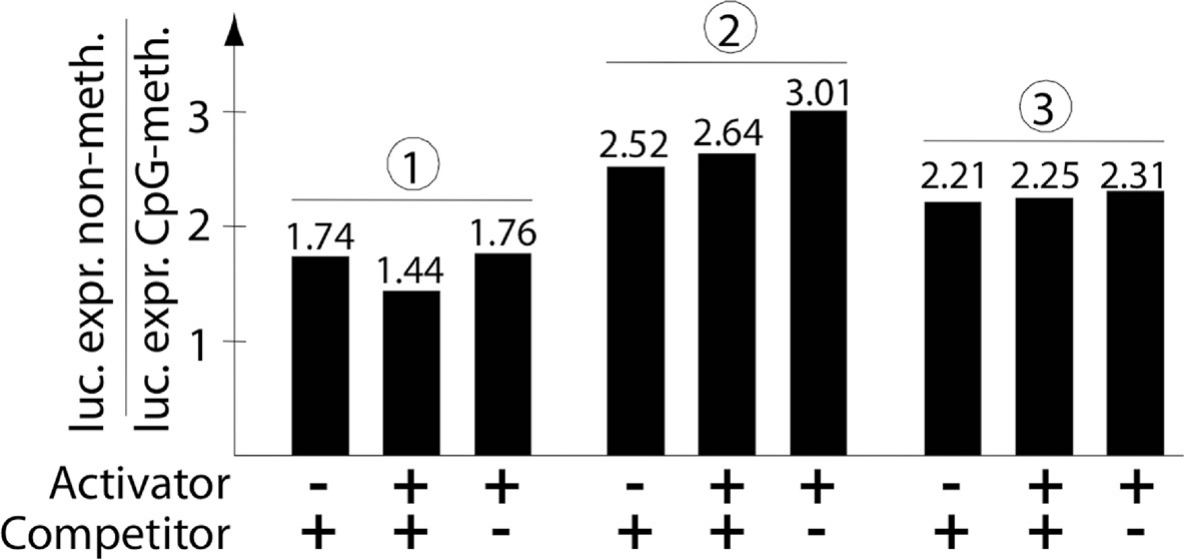
***In vivo *binding of *SB *transposase.** Untreated or CpG-methylated p5SB-luc reporter plasmids were co-transfected with/without the activator plasmid pc-SB(123)-AD and with/without a full-length *SB* transposase competitor into HeLa cells. After 48 h luciferase expression was determined. To compare the effect of CpG methylation, ratios were calculated by dividing the value for luciferase expression with untreated donor plasmids by the value for luciferase expression with CpG-methylated donors. Three independent experiments are shown. No statistically significant differences were obtained.

Transposition is a multistep process consisting of: (i) binding the transposase to the TIRs, (ii) synaptic complex formation, (iii) transposon excision, (iv) target capture and (v) transposon integration. Each of the studied IR/DR TEs contains at least one CpG site in the TIRs (*SB*: 2, *FP*: 1, *Minos*: 11). In order to address whether CpG methylation affects binding of the *SB* transposase to its binding sites in the TIRs, we applied a one-hybrid assay [25] to cultured cells. The reporter plasmid contained a luciferase gene behind a minimal promoter and four *SB* transposase binding sites upstream of the promoter. Untreated or CpG-methylated reporter plasmids were co-transfected with (i) the transcriptional activator plasmid expressing N123-AD containing the N-terminal DNA-binding domain of the *SB* transposase fused to a VP16-transactivation domain that induces luciferase expression upon TBS binding and (ii) a plasmid expressing full-length *SB* transposase that binds TBS, but does not transactivate luciferase expression, and thus acts as a competitor of N123-AD. The activator induced luciferase expression >350-fold, and co-expression of the competitor reduced luciferase expression up to tenfold for both untreated and CpG-methylated reporters (Additional file 1: Table S1). CpG methylation of the reporter plasmid reduced luciferase expression, presumably by promoter silencing (Figure 3). However, the extent of reduction was always similar in the presence or absence of the activator and the competitor (Figure 3). We conclude that the *SB* transposase does not have either reduced or enhanced affinity for CpG-methylated DNA.

### Methylation of CpG sites in transposon inverted repeats is not required for enhanced transposition

**Figure 4:**
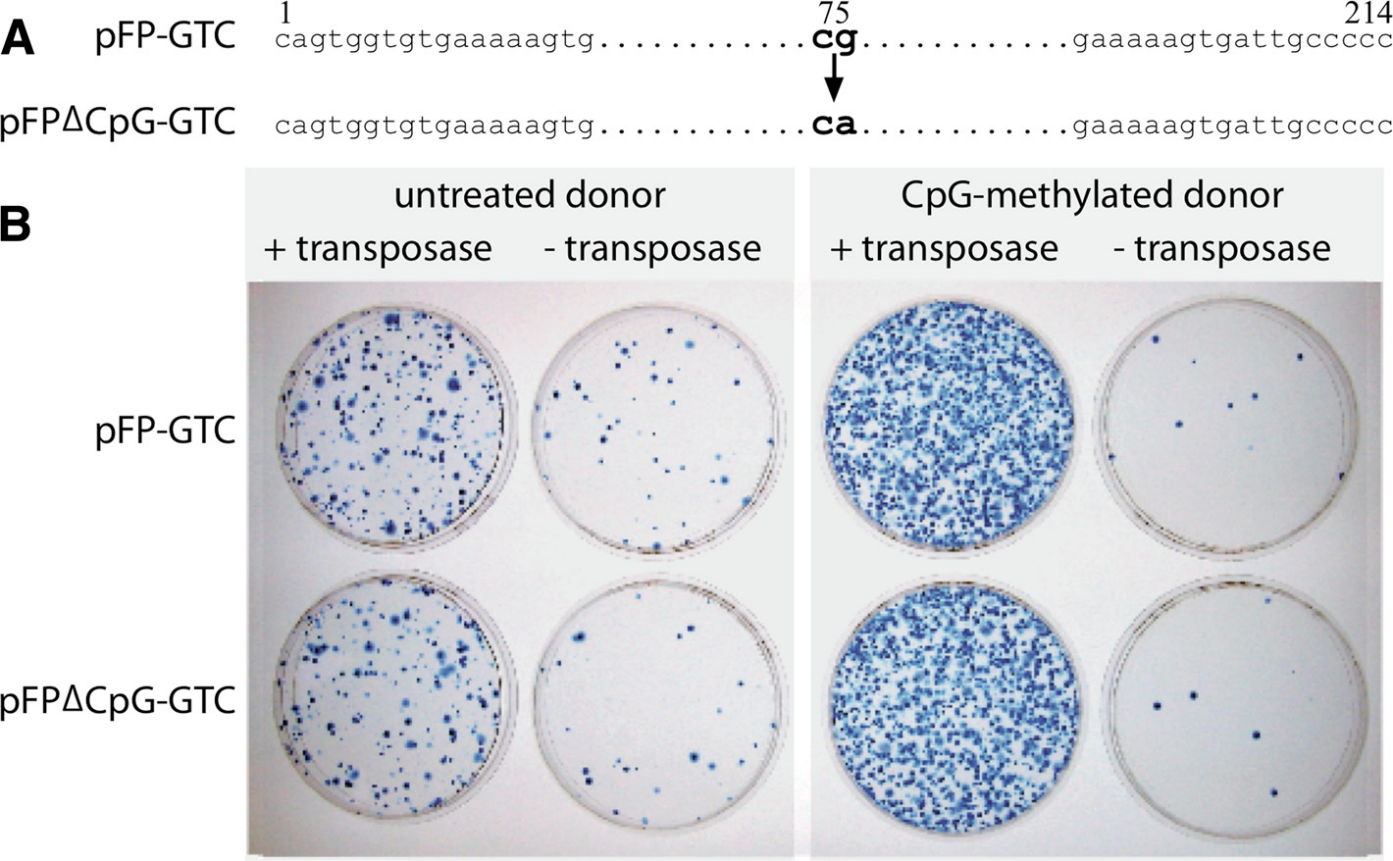
**Transposition of CpG-free *FP *TIRs.** (**A**) CpG sites inside the *FP* TIRs were mutated to obtain pFPΔCpG-GTC. (**B**) Transposition assay of CpG-methylated or untreated pFPΔCpG-GTC and pFP-GTC was performed in HeLa cells in the presence or absence of transposase. *FP*: *Frog Prince*; TIR: terminal inverted repeat.

Methylation of CpG sites in the transposon TIRs may affect the affinity of DNA-binding proteins [26], for example, it might increase binding of an enhancer or reduce binding of an inhibitor of transposition, thereby enhancing transposition. Both *SB* and *Minos* contain CpG sites in their TBSs and, therefore, mutagenesis of the TBS sequence is expected to drastically affect or abolish transposase binding. However, *FP* has only one CpG site in its TIRs outside the TBSs (plus 435 additional CpG sites distributed all across the rest of the plasmid). We mutated the CpG site at both TIRs of *FP* to a CpA sequence that cannot be methylated, and compared the transpositional activity of the mutant transposon to the wild-type transposon in the absence and presence of CpG methylation. No differences in transposition were detected either for untreated or for CpG-methylated donor plasmids (Figure 4). We conclude that it is not a particular CpG site inside the TIRs, but rather the global CpG content of the transposon plasmid that is responsible for the observed enhancing effect of CpG methylation.

### CpG methylation enhances *Sleeping Beauty* transposon excision *in vivo*

**Figure 5:**
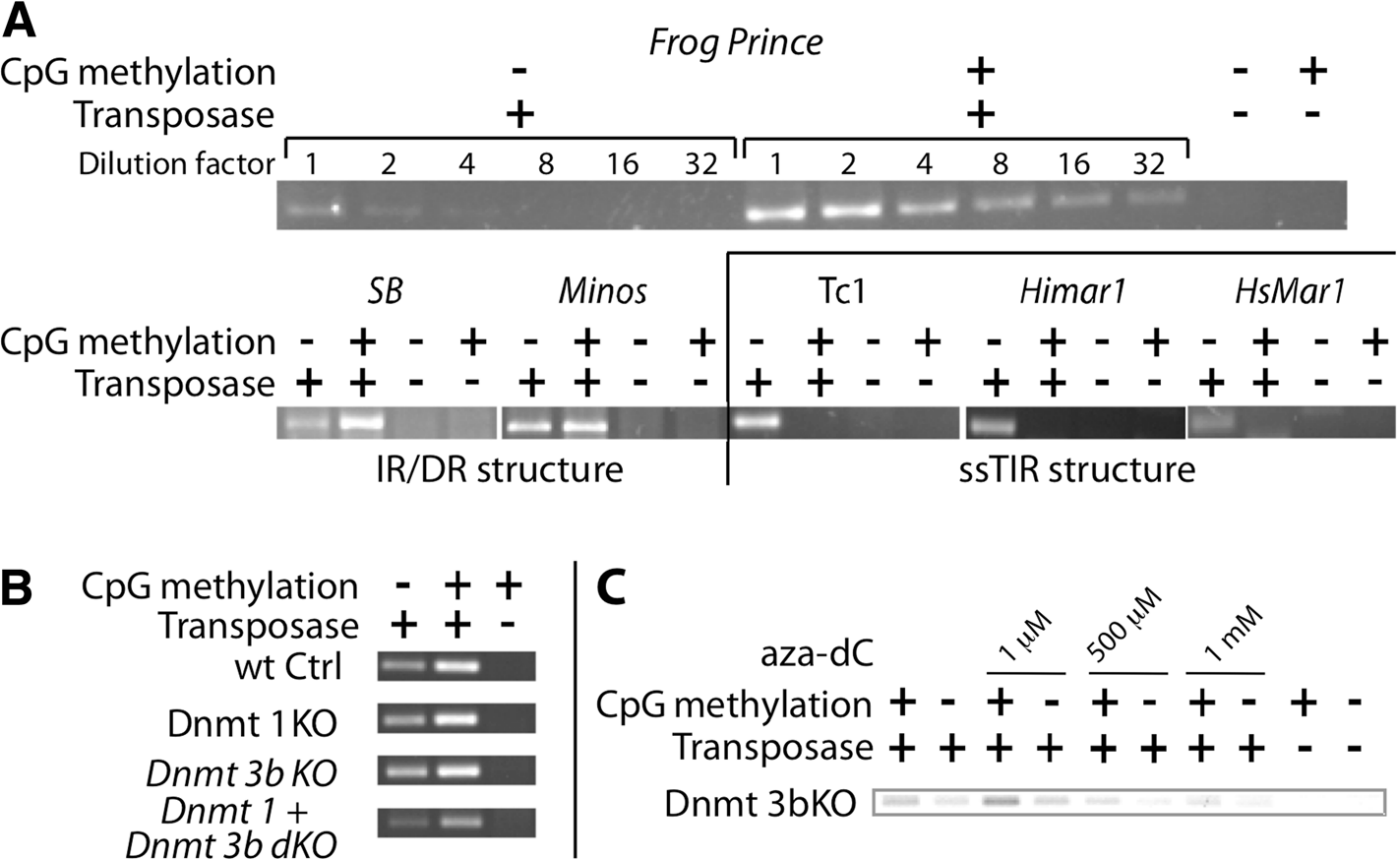
**Excision activity upon CpG methylation.** CpG-methylated or untreated donor plasmids were co-transfected with/without a helper plasmid; 48 h post-transfection plasmids were extracted, purified and DNA amounts were normalized. After two rounds of nested PCR, the product band intensity represents the relative efficiency of transposon excision. (**A**) Excision activity of different TEs in HeLa cells. Upper panel: Dilutions of PCR input DNA for quantification of *FP* excision. (**B**) Excision of *SB* in different HCT116 knockout cell lines. (**C**) *SB* excision in HCT116 Dnmt3b knockout cells. Increasing amounts of aza-deoxycytidine (aza-dC) block *de novo* CpG methylation. aza-dC: aza-deoxycytidine; *FP*: *Frog Prince*; *SB*: *Sleeping Beauty*; PCR: polymerase chain reaction; TE: transposable element.

In order to identify the step(s) at which CpG methylation influences transposition, we subjected the six elements to a PCR-based excision assay. CpG-methylated or untreated donor plasmids were co-transfected with the respective helper plasmids into cultured HeLa cells and were re-isolated 48 h post-transfection. Amplification of transposon excision sites of the donor plasmids via nested PCR produces a 308-bp-long amplicon, whose band intensity in gel electrophoresis was used as measure of transposon excision. Consistent with the effect on transposition, excision of IR/DR-elements *SB*, *FP* and *Minos* was enhanced by CpG methylation (Figure 5A). By serial dilution of PCR input DNA, we quantified the increase of excision efficiency for *FP*, which is enhanced at least 16-fold by CpG methylation. This is in accordance with the 20-fold increase in overall transposition upon CpG methylation obtained in the colony-based assay. Again, the group of simple-structured TIR (ssTIR) elements showed no increase in excision activity; in contrast, excision was significantly lower or even undetectable following CpG methylation. Thus, the enhancing effect of CpG methylation on transposon excision is limited to the elements of the IR/DR group.

Transiently transfected plasmids can become CpG-methylated in the cell *de novo*[27]. CpG methylation in mammals is catalyzed by three DNA-methyltransferases (Dnmts): Dnmt1 is responsible for the maintenance of CpG methylation patterns, and Dnmt3a and Dnmt3b perform *de novo* CpG methylation. We tested the excision activity of untreated and CpG-methylated *SB* transposon donors in HCT116 knockout/knockdown cell lines lacking either Dnmt1 or Dnmt3b or both [28,29] by PCR-based excision assay (Figure 5B). All cell lines supported transposon excision. However, in these cells Dnmt3a is still active and could provide *de novo* CpG methylation of transfected donor plasmids. Thus, to inhibit the activity of Dnmt3a, we used the chemical aza-deoxycytidine, which is a known inhibitor of cellular *de novo* CpG methylation. Aza-deoxycytidine was added to cultured, HCT116-derived Dnmt3b knockout cells in a range of 1 μM to 1 mM two days prior to co-transfection of the transposon plasmids. The PCR-based excision assay (Figure 5C) showed that transposon excision is still detectable at high concentrations of aza-deoxycytidine. Even though PCR band intensity became weaker at increasing aza-deoxycytidine concentrations, this is likely due to the cytotoxic effects of aza-deoxycytidine, which is accompanied by reduced cell survival and cell growth. The enhancing effect of *in vitro* CpG methylation was not affected by aza-deoxycytidine treatment. We conclude that host-mediated *de novo* CpG methylation is not required for transposition.

### CpG-methylated transposon plasmids are associated with condensed chromatin

**Figure 6:**
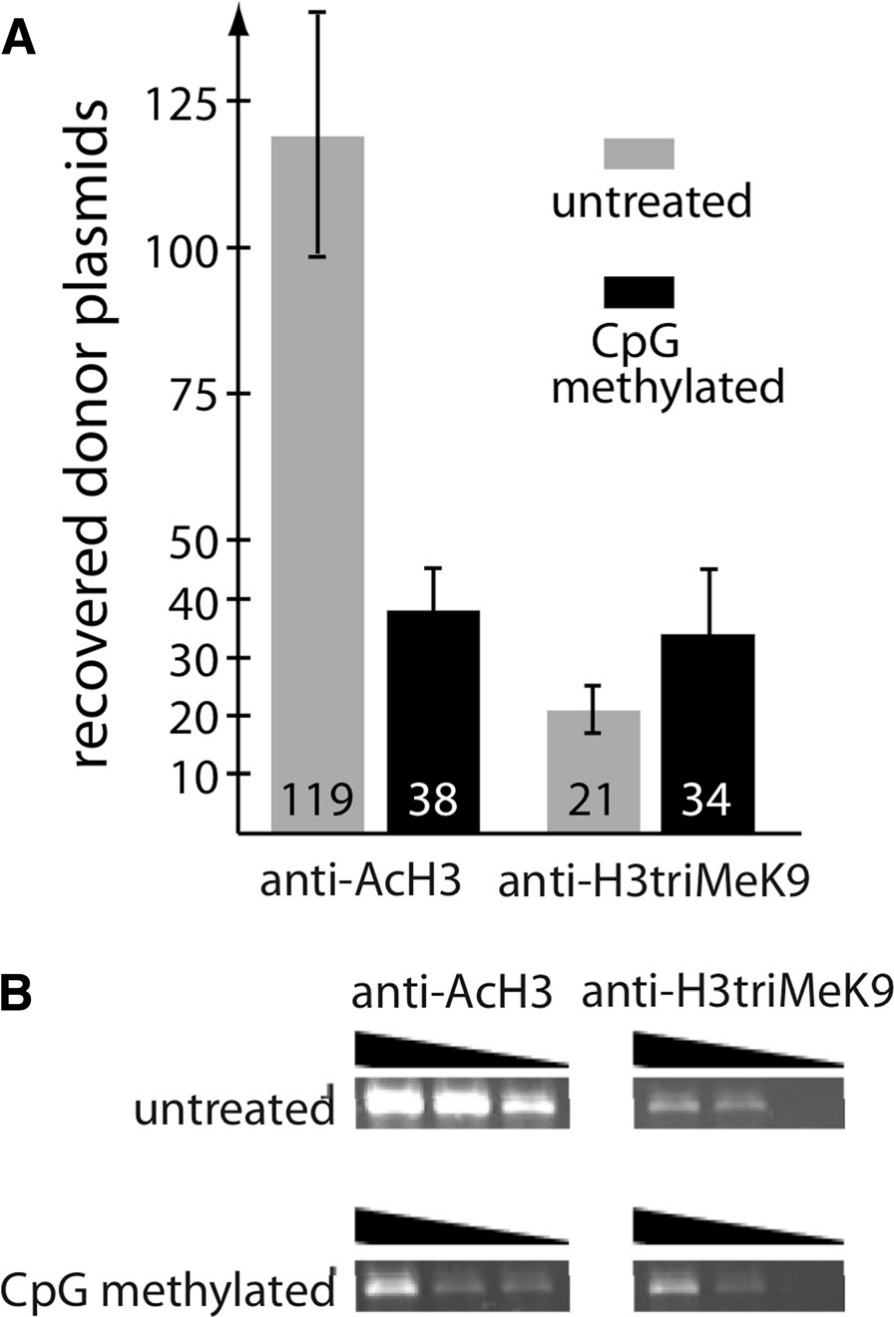
**CpG-methylated transposon plasmids are associated with condensed chromatin.** CpG-methylated or untreated pFP-GTC donor plasmids were transfected into HeLa cells, cross-linked, and precipitated with anti-acetylated histone H3 (anti-AcH3) antibodies indicating euchromatin or anti-trimethylated histone H3 lysine 9 (anti-H3triMeK9) antibodies indicating heterochromatin. DNA was purified and quantified by *E. coli* transformation (**A**) or by semi-quantitative PCR after serial dilutions of input DNA (**B**). anti-AcH3: anti-acetylated histone H3; anti-H3triMeK9: anti-trimethylated histone H3 lysine 9; PCR: polymerase chain reaction.

As CpG methylation did not seem to have a direct influence on transposition, we hypothesized that it affects the transposition process indirectly, by inducing heterochromatin formation and persistence. Indeed, genomic, CpG-methylated transposons were shown to be associated with heterochromatin [17]. We utilized a chromatin immunoprecipitation (ChIP) assay to investigate chromatin structure on transposon donor plasmids. CpG-methylated and non-methylated pFP-GTC donor plasmids were transfected into HeLa cells, cross-linked and immunoprecipitated using antibodies against acetylated histone H3 (a marker for open chromatin) or against trimethylated H3K9 (a marker for condensed heterochromatin). The co-precipitated DNA was purified and analyzed by (i) semi-quantitative PCR and (ii) transformation of recovered donor plasmids into *Escherichia coli* and subsequent colony counting (Figure 6).

As quantified by bacterial transformation, anti-acetylated histone H3 (anti-AcH3) antibodies precipitated threefold less CpG-methylated transposon plasmids than non-methylated transposon plasmids, suggesting an enrichment of non-methylated plasmids in open chromatin (Figure 6A). Conversely, immunoprecipitation with anti-trimethylated histone H3 lysine 9 (anti-H3triMeK9) antibodies resulted in reduced recovery of non-methylated plasmids compared to CpG-methylated plasmids, implying an enrichment of condensed chromatin status for CpG-methylated plasmids. PCR analyses of the immunoprecipitated DNA samples showed, in accordance with the results obtained by bacterial transformation, a relative enrichment of non-methylated plasmids in the euchromatic fraction (precipitation with anti-AcH3 antibody) and a relative enrichment of methylated plasmids in the heterochromatic fraction (precipitation with anti-H3triMeK9 antibody) (Figure 6B). We conclude that CpG-methylated plasmids are associated with condensed chromatin.

### Precomplexing transposon DNA with protamine enhances *Sleeping Beauty* transposition

To test if DNA condensation is an underlying structural determinant of enhanced transposition in our experiments, we sought to examine the effect of condensation introduced into transposon DNA by means other than CpG methylation. We opted to package and condense transposon donor plasmids using protamine. Protamines are small, basic, arginine-rich peptides that largely replace histones in sperm chromatin, and package DNA into the most condensed eukaryotic DNA known [30].

**Figure 7:**
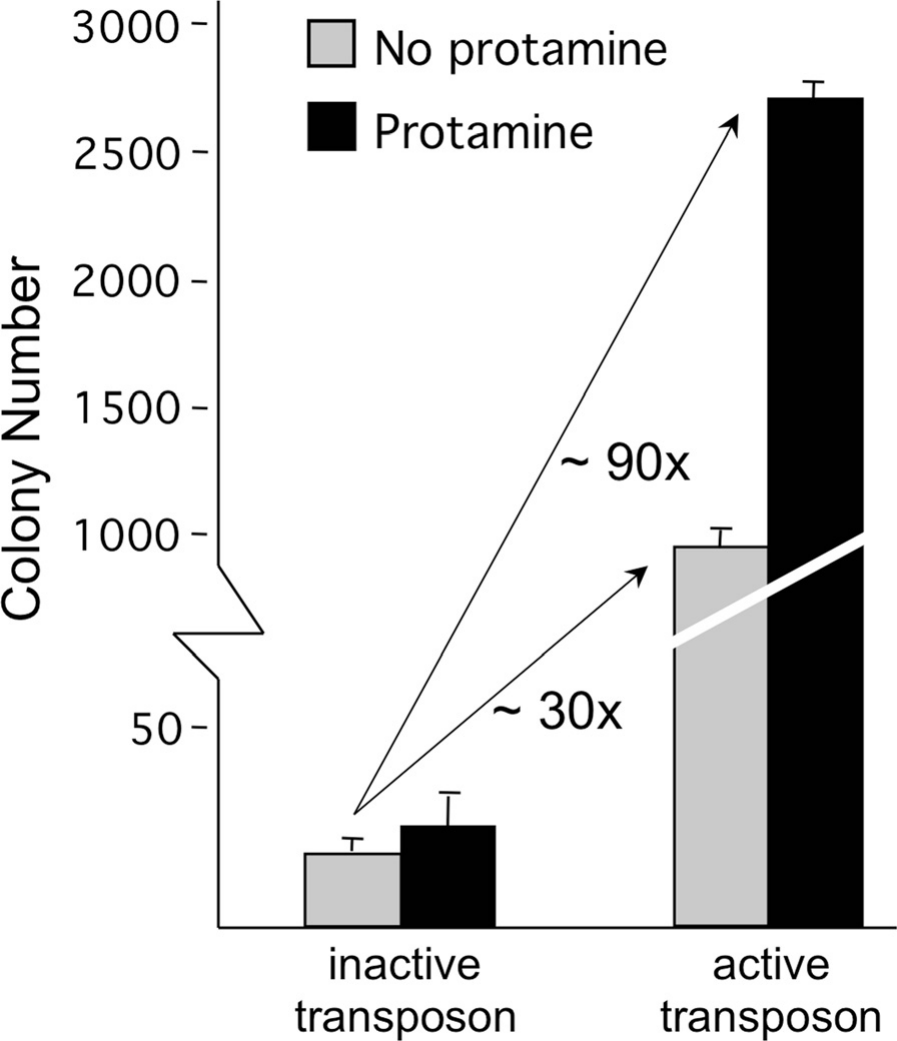
**Protamine enhances *SB *transposition.** Transpositionally competent as well as incompetent (negative control) *SB* transposon plasmids were precomplexed with protamine *in vitro*, followed by transfection into transgenic HeLa cells stably expressing the *SB* transposase. The transfected cells have undergone antibiotic selection to allow a quantitative measurement of transposition efficiencies represented by antibiotic-resistant colony numbers. *SB*: *Sleeping Beauty*.

Antibiotic-resistance-gene-containing *SB* transposon donor plasmids were pre-incubated with protamine, and transfected into human HeLa cells expressing *SB* transposase, followed by antibiotic selection and counting of resistant cell colonies. Pre-incubation of *SB* transposons with protamine resulted in an approximately threefold enhancement of transposition compared to uncondensed plasmids (Figure 7). The enhancing effect of protamine on stable gene transfer was only manifested with transposition-competent vectors; treatment of transpositionally incompetent vectors with protamine apparently had no effect on colony numbers (Figure 7). We conclude that condensation of transposon DNA has an enhancing effect on *SB* transposition.

## Discussion

The original finding that CpG methylation enhances *Sleeping Beauty* transposition [17] was very surprising, as CpG methylation has been known to play a crucial role in the cellular defense against TEs. We now extend these observations to other members of the *Tc1*/*mariner* superfamily, and show that this phenomenon is not restricted to *SB*, but seems to be an intrinsic feature associated with the characteristic IR/DR structure of the *SB*, *Frog Prince* and *Minos* elements (Figure 2).

We tested several hypotheses that provided possible explanations for the enhancing effect of CpG methylation on transposition. It is formally possible that CpG methylation is a prerequisite for transposition of *SB*, *FP* and *Minos*. In this case donor plasmids that are CpG-methylated *in vitro* would have a head start against non-methylated donors, which would need to be methylated by cellular factors following transfection. We tested transposition of non-methylated donor plasmids in Dnmt1 and/or Dnmt3b knockout/knockdown cell lines and in the presence of aza-deoxycytidine, which blocks *de novo* CpG methylation. Transposition was as efficient under these conditions as in wild-type or untreated cells (Figure 5), thus we conclude that CpG methylation is not required for transposition.

An alternative hypothesis predicts that one (or several) particular CpG sites inside the TIRs might affect CpG methylation. An enhancer of transposition might possibly be attracted by methylated CpG sites or, alternatively, a potential repressor might be distracted. However, a mutated *FP* donor plasmid with depleted CpG sites in both TIRs was as efficient in transposition as the wild-type transposon (Figure 4). Thus, CpG sites inside the TIRs do not seem to be responsible for the binding of any transposition-enhancing factor or the blocking of any transposition repressor.

Binding of the *SB*, *FP* and *Minos* transposases might directly be supported by CpG sites inside the TBSs, the TIRs or close to them. Alternatively, CpG methylation could increase transposition indirectly; for example by the formation of heterochromatin following binding of methylated CpG sites by the HP1 or MeCP2 (a protein that binds specifically to methylated DNA) proteins [31,32]. Yusa *et al.*[17] tested *SB* transposase binding to TBSs *in vitro* and did not detect any influence from CpG methylation. In line with these previous observations, the expression of luciferase as a reporter for TBS binding in an *in vivo* one-hybrid assay was affected by CpG methylation to the same extent in all background controls, binding experiments and under competition conditions (Figure 3). We thus conclude that CpG methylation had neither a direct nor an indirect effect on *SB* transposase binding.

**Figure 8:**
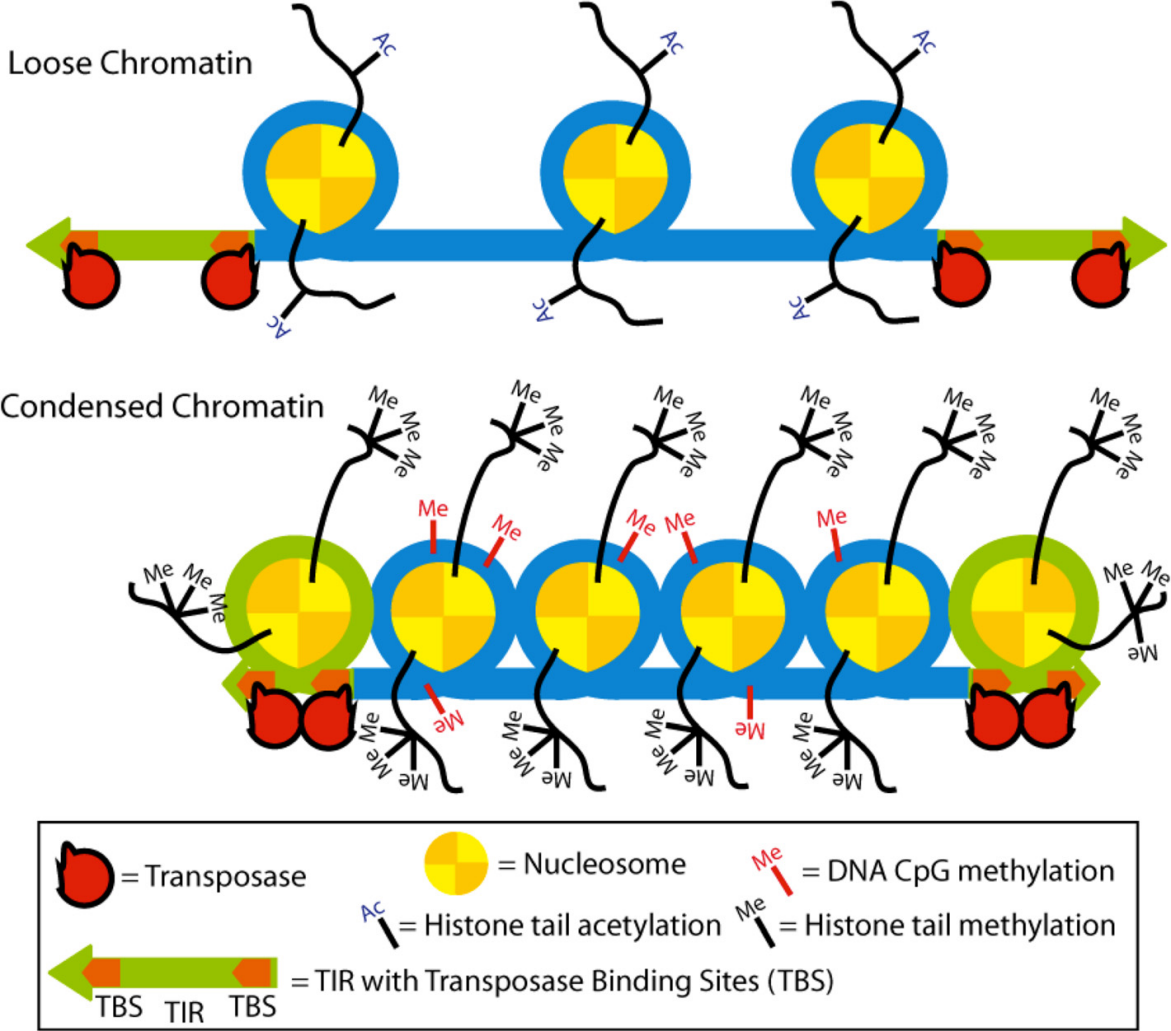
**A model of a molecular mechanism of enhanced synaptic complex assembly by condensed DNA packed into heterochromatin.** CpG methylation induces heterochromatin condensation whereby the TBSs inside the TIRs will come into close proximity. Hence, the formation of transposase dimers is improved, which subsequently enhances the formation of synaptic complexes. TBS: transposase binding site; TIR: terminal inverted repeat.

Our findings suggest an indirect effect of CpG methylation on transposition rather than a direct influence. Because (i) enhancement of CpG methylation is detectable at the transposon excision step (Figure 5), (ii) CpG methylation was found to induce the formation of a condensed chromatin structure (heterochromatin) (Figure 6), and (iii) DNA compaction by protamine was found to enhance transposition (Figure 7), we propose a model, in which CpG methylation and subsequent chromatin condensation aids synaptic complex formation. Indeed, transposition critically depends on the formation of a synaptic complex that is built up of the transposon TIRs, transposase proteins and additional, host-encoded proteins. It is formally possible that CpG methylation-induced chromatin condensation brings the TIRs closer together, thereby promoting synaptic complex assembly. However, our studies on several, closely related *Tc1*/*mariner*-elements revealed the selective importance of TIR structure for the observed sensitivity for CpG methylation. Namely, only the *SB*, *FP* and *Minos* elements were responsive to CpG methylation; these IR/DR elements share a common structure of two TBSs per TIR (Figure 1). Heterochromatin formation results in tight packaging of DNA and histones. As a result, DNA sites that are usually far away from each other; for example, the two TBSs inside one TIR, might be brought closer together (Figure 8). The physical proximity of the inner and outer TBSs might assist the formation of transposase dimers as soon as they bind, thereby facilitating the formation of a catalytically active synaptic complex.

## Conclusions

CpG methylation/heterochromatin is one of the regulatory mechanisms that silence and inhibit TE activity. The potential of TEs to escape a regulatory mechanism imposed by the host is a strong evolutionary advantage (at least for the transposon). Assuming that the transposase source is provided by a transcriptionally active element located in euchromatin, host-cell induced CpG methylation/heterochromatin-based silencing of TEs can be offset by higher transposition efficiency out of condensed chromatin, thereby constituting a potential mechanism for *SB* and other, similar-structured transposons to escape CpG methylation-mediated silencing.

## Methods

### Plasmid construction

A 200 bp-minivariant of *Himar1* (GenBank #U11644) was amplified out of pMM2611 [33] via PCR using the primer *Himar1*/IR (TAACAGGTTGGCTGATAAGTCCCC). A 1.6 kb fragment of *Tc1* (GenBank #X01005) was amplified out of pTc1_Ex/01 with primer Tc1/IR (TACAGTGCTGGCCAAAAAGATATCC). The PCR products were purified, phosphorylated and ligated into a SmaI digested, dephosphorylated vector pUC19. After transformation in *E. coli* DH10B cells, plasmids were isolated, test digested and sequenced. pUC19-*Himar1* was SmaI digested, pUC19-*Tc1* was StyI digested; both were dephosphorylated and ligated with the Klenow-treated gene trap cassette (GTC) (HindIII-NotI-XmnI digest of plasmid Δ170_CMV_zeo#1) [20]. After transformation in *E. coli* DH10B, plasmids were prepared and control digested to verify the expected plasmid layout. For *Minos* (GenBank #X61695) the plasmid pMiLR*neo*[34] was HindIII-NotI digested and ligated with the formerly described HindIII-NotI fragment of the GTC. The TIRs harboring the GTC were amplified out using PCR primer *Minos*/IR (TACGAGCCCCAACCACTATTAATTC). The PCR product was purified and ligated into SmaI digested pUC19. The product was checked by test digestion and sequencing. pSB-GTC (= GT/neo_CMV/zeo #2), derived from pT/neo [35], pFP-GTC (= pFP/GT-neo) [20] and pHsMar1-GTC and pHsmar1-GTCrev [24] were provided by C Miskey. The plasmids containing the transposases have been previously published: *Tc1*: pCMV/Tc1 [36], *Himar1*: pCMV/Himar3x [36], p*SB10*: [35], *Minos*: pJGD/ILMi [37], *FP*: pFV-FP [20], *Hsmar1*: p*Hsmar1*[24]. pFPΔCpG-GTC: *FP* TIRs on plasmid pFP-GTC were mutated using the Stratagene QuikChange Multi Site Directed Mutagenesis Kit following the manufacturer’s instructions with phosphorylated primers *FP*/IR_CpG_delete_syn (P-TGTTTGTCACACTTAA-GTGTTT**CA**GAACATCAAA-CCAATTTAAACAATAG) and *FP*/IR_CpG_delete_anti (P-CTATTGTTTAAATTGGTTTGATGTTC**TG**AAACA-CTTAAGTGTGACAAACA). The mutated plasmid pFPΔCpG-GTC was transformed in QuikChange XL1-Blue Supercompetent Cells (Stratagene), purified and sequenced.

### *In vitro* CpG methylation

Donor plasmids were CpG-methylated by SssI CpG methylase (NEB), and purified with the Qiagen PCR purification kit. Complete methylation was tested by control digests using methylation-sensitive restriction enzymes NotI (pMiLR*geo*) or SalI (all others), and compared to a control digest of the respective untreated donor plasmids.

### Cell culture, transfections and DNA condensation by protamine

HeLa cells were cultured in DMEM with 4.5 g/L glucose and 110 mg/L pyruvate supplemented with 10% fetal calf serum (FCS) and an antibiotic/antimycotic cocktail (DMEM+/+) at 37°C. Cells were passaged via trypsinization with 1:5 dilution of 0.5% trypsin in 5.3 mM ethylenediaminetetraacetic acid (EDTA). Transfections were done at 60% to 80% cell confluency in DMEM without antibiotics (DMEM+/−) with the Fugene6 transfection reagent (Roche). For transposition assays, 6 × 10^5^ cells were transfected with CpG-methylated or untreated donor plasmids (500 ng) together with transposase expression plasmids (50 ng). Complexing with protamine sulfate was done as described previously [38] by pre-incubating 500 ng plasmid DNA with 1 μg protamine sulfate (Sigma-Aldrich) for 15 min at room temperature, followed by the addition of Fugene6 as described above.

### Excision PCR

Cells (6 × 10^5^) were transfected with equal amounts of CpG-methylated or untreated donor plasmids plus equal amounts of transposase-carrying helper or control plasmids. Typically 250 ng or 500 ng of donor plasmid was used and 50 ng of helper plasmid. Two days after transfection, the cells were harvested, plasmid DNA was prepared with the Qiagen Miniprep kit in a volume of 50 μL. A 1:10 dilution of 1 μL of the extracted DNA served as template for input normalization PCR utilizing primers Amp-For (TGCACGAGTGGGTTACATCGAACT) and Amp-Rev (TTGTTGCCATTGCTACAGGCATCG). Normalized DNA amounts served as templates for nested PCR utilizing pUC2 (GCGAAAGGGGGATGTGCTGCAAGG) and pUC5 (TCTTTCCTGCGTTATCCCCTGATTC) in the first, and pUC19-3F (GTTTTCCCAGTCACGACGTT) and pUC19-3R (TGTGGAATTGTGAGCGGATA) in the second PCR. PCR protocol: 95°C 3 min, 30 cycles of 95°C 30 s, 58°C 20 s, 64°C 10 s, and 72°C 2 min. The PCR products were subjected to gel electrophoresis.

### Chemical block of CpG methylation

Two days prior to transfection cells were treated with different amounts of 5-aza-2′-deoxycytidine (aza-dC), ranging from 1 μM to 1 mM. Aza-dC addition was repeated daily after previous medium exchange. Then, 6 h before transfection, aza-dC was removed from the cells and added again 6 h post-transfection.

### One-hybrid assay

HeLa cells (6 × 10^5^) were transfected with 300 ng CpG-methylated or untreated p5SB-luc reporter plasmid, 90 ng pc-SB(123)-AD activator plasmid [25] and 1.1 μg pCMV-SB10 competitor plasmid (or filled with adequate control plasmids). Two days later, transfected cells were washed, treated with CCLR buffer (5× buffer: 125 mM Tris*H_3_PO_4_ pH 7.8, 10 mM dithiothreitol (DTT), 10 mM 1,2 cyclohexane-diaminetetra-acetic acid (CDTA), 50% glycerol, 5% Triton X-100), incubated for 15 min on ice and vortexed for 15 s. Cell debris was pelleted for 2 min with 12,000*g* at 4°C. 30 μL of the supernatant was combined with 100 μL luciferase buffer (20 mM tricine-NaOH pH7.8, 1.07 mM Mg(CO_3_)_4_ Mg(OH)_2_*5H_2_O, 2.67 mM MgSO_4_, 0.1 mM EDTA, 470 μM luciferin, 530 μM ATP, 33.3 mM DTT), vortexed briefly and measured in the luminometer.

### Chromatin immunoprecipitation

Cells (4×10^6^) were transfected in either four 5-cm or one 10-cm culture dish with approximately 3 μg CpG-methylated or non-methylated pFP-GTC donor plasmid. 24 h after incubation, 37% formaldehyde was added to the medium to an end concentration of 1%. The culture dishes were put into a plastic bag, sealed and incubated for 10 min at 37°C. After removal of the medium and two washing steps with ice-cold PBS containing protease inhibitors (1 mM PMSF, 1 μg/mL aprotinin, 1 μg/mL pepstatin A), the cells were scraped, transferred into 1.5 mL Eppendorf tubes and centrifuged for 4 min at 4°C at 2,000*g*. The pellet was resuspended in 400 μL lysis buffer [1% sodium dodecyl sulfate (SDS), 10 mM EDTA, 50 mM Tris–HCl pH 8.1, protease inhibitors]. Each sample was split into 2× 200 μL samples, incubated for 10 min on ice and centrifuged for 10 min at 4°C at 13,000 rpm. Supernatants were transferred to fresh 2 ml Eppendorf tubes and 1,800 μL ChIP dilution buffer (0.01% SDS, 1.1% Triton X-100, 1.2 mM EDTA, 16.7 mM Tris–HCl pH 8.1, 167 mM NaCl, protease inhibitors) and 80 μL salmon sperm DNA/Protein A agarose-50% slurry (0.2 mg/mL sonicated salmon sperm DNA, 0.5 mg/mL BSA, approximately 1.5 mg/mL recombinant Protein A, 0.05% sodium azide) were added. The Eppendorf tubes were incubated for 30 min on a rotating plate at 4°C. The agarose was pelleted using brief centrifugation and the supernatants were transferred to new tubes. Polyclonal antibodies anti-acetylated histone H3 (anti-AcH3) (provided with the ChIP Kit, Upstate #06-599, Lot 27610) or anti-trimethylated histone H3 lysine 9 (anti-H3triMeK9) (Abcam #ab8898) were added in varying amounts (4 μL to 10 μL) and incubated overnight at 4°C on a rotating plate. The next day, 60 μL of salmon sperm DNA/Protein A agarose-50% slurry was added and incubated for 1 h at 4°C with rotation. The agarose was pelleted at 900 rpm for 1 min at 4°C and washed with low salt immune complex wash buffer (0.1% SDS, 1% Triton X-100, 2 mM EDTA, 20 mM Tris–HCl pH 8.1, 150 mM NaCl), high salt immune complex wash buffer (0.1% SDS, 1% Triton X-100, 2 mM EDTA, 20 mM Tris–HCl pH 8.1, 500 mM NaCl), LiCl immune complex wash buffer [0.25 M LiCl, 1% Nonidet P-40 (NP40), 1% deoxycholate, 1 mM EDTA, pH 8.0] and twice with TE buffer (10 mM Tris–HCl, 1 mM EDTA, pH 8.0). The chromatin was eluted by the repeated addition of 250 μL elution buffer (1% SDS, 0.1 M NaHCO_3_) to the pellet, vortexing and 15 min incubation followed by pelleting the agarose and saving the supernatant in a fresh 1.5 mL tube. To the combined (500 μL) eluates, 20 μL 5 M NaCl was added and heated for 4 h at 65°C to reverse cross-linking (samples were stored at this point overnight at −20°C). Using 10 μL 0.5 M EDTA, 20 μL 1 M Tris–HCl pH 6.5 and 2 μL proteinase K, histones and other DNA-bound proteins were digested for 1 h at 45°C. The remaining residues were extracted twice with phenol/chloroform and the DNA was precipitated with isopropanol (standard protocol). The DNA was taken up in H_2_O and re-purified by dialysis. For the quantification of plasmids, the extracted DNA was either transformed into electrocompetent *E. coli* DH10B or used in a semi-quantitative PCR (primer Amp-For and Amp-Rev). Transformed cells were plated on ampicillin/zeocin/LB (Luria-Bertani) agar and incubated overnight at 37°C. Colonies were counted the next morning.

## Abbreviations

anti-AcH3: Anti-acetylated histone H3; anti-H3triMeK9: Anti-trimethylated histone H3 lysine 9; aza-dC: aza-deoxycytidine (5-aza-2′-deoxycytidine); BSA: Bovine serum albumin; CDTA: 1,2 cyclohexane-diaminetetra-acetic acid; ChIP: Chromatin immunoprecipitation; DTT: Dithiothreitol; DMEM: Dulbecco’s modified Eagle’s medium; Dnmt: DNA-methyltransferase; EDTA: Ethylenediaminetetraacetic acid; ES: Embryonic stem; FCS: Fetal calf serum; FP: *Frog Prince*; GTC: Gene trap cassette; HP1: Heterochromatin protein 1; LB agar: Luria-Bertani agar; NP40: Nonidet P-40; PCR: Polymerase chain reaction; SB: *Sleeping Beauty*; SDS: Sodium dodecyl sulfate; ssTIR: Simple-structured TIR; TBS: Transposase binding site; TE: Transposable element; TIR: Terminal inverted repeat.

## Competing interests

The authors declare that they have no competing interests.

## Authors’ contributions

TJ designed and performed all of the experiments in the laboratory of ZIv. ZIv designed and coordinated the study. ZIz participated in the design and coordination of the study. CM participated in both design and execution of some of the experiments. TJ and ZIv wrote the manuscript. All authors read and approved the final manuscript.

## Supplementary Material

Additional file 1: Table S1 One-hybrid assay reads. Reporter, activator and competitor plasmids were co‒transfected into HeLa cells. Cells were harvested 48 h post‒transfection, and protein extracts were used in luciferase assays. Three repetitions are shown. Click here for file
