# Supplementary material for: Regulation of DNA transposition by CpG methylation and chromatin structure in human cells
Source: Mob DNA. 2013 May 15;4:15. doi: 10.1186/1759-8753-4-15 (PMC3680223; doi:10.1186/1759-8753-4-15)
Supplement: Additional file 1: Table S1 — One-hybrid assay reads. Reporter, activator and competitor plasmids were co‒transfected into HeLa cells. Cells were harvested 48 h post‒transfection, and protein extracts were used in luciferase assays. Three repetitions are shown. [file 1759-8753-4-15-S1.pdf]

Experiment	Activator	Competitor	Methylation	Average Luc values
1	-	+	-	1476
	+	+	-	2342
	+	-	-	21643
	-	+	+	849
	+	+	+	1629
	+	-	+	12289
	-	-	-	44
2	-	+	-	2431
	+	+	-	6488
	+	-	-	17297
	-	+	+	966
	+	+	+	2458
	+	-	+	5748
	-	-	-	54
3	-	+	-	1693
	+	+	-	4479
	+	-	-	25389
	-	+	+	766
	+	+	+	1995
	+	-	+	10998
	-	-	-	84

Supplementary Table 1. One-hybrid assay reads. Reporter, activator and competitor plasmids were co-transfected into HeLa cells. Cells were harvested 48 hrs post-transfection, and protein extracts were used in luciferase assays. Three repetitions are shown.
